# Comprehensive Meta-Analysis of Differentially Expressed Proteins in Cerebrospinal Fluid Associated with Multiple Sclerosis

**DOI:** 10.3390/ijms26136171

**Published:** 2025-06-26

**Authors:** Elif Sakiz, Elnaz Amanzadeh Jajin, Liza Cubeddu, Roland Gamsjaeger, Timucin Avsar

**Affiliations:** 1 School of Science, Western Sydney University, Sydney, NSW 2751, Australia; e.sakiz2@westernsydney.edu.au (E.S.); l.cubeddu@westernsydney.edu.au (L.C.); r.gamsjaeger@westernsydney.edu.au (R.G.); 2 Neuro-Oncology Laboratory, School of Medicine, Bahcesehir University, Istanbul 34734, Türkiye; 3 Functional Neurosurgery Research Centre, Shohada Tajrish Comprehensive Neurosurgical Centre of Excellence, Shahid Beheshti University of Medical Sciences, Tehran P.O. Box 1988873554, Iran; elnazamanzadehjajin@gmail.com; 4 Department of Medical Biology, Bahçeşehir University School of Medicine, Istanbul 34734, Türkiye

**Keywords:** cerebrospinal fluid, CSF, meta-analyses, multiple sclerosis, neurodegeneration, proteomics

## Abstract

To advance our understanding of multiple sclerosis (MS), accurate identification of protein expression profiles as biomarkers for MS in cerebrospinal fluid (CSF) is critical. However, proteomic studies investigating MS have yielded inconsistent findings due to variability in sample sizes, diagnostic criteria, and data processing methods. We aimed to tackle these challenges by performing a thorough meta-analysis of proteomics datasets sourced from multiple independent studies. We conducted a thorough database search to gather all relevant studies using appropriate keywords. We screened articles using defined inclusion and exclusion criteria, and finally, six studies were included. We retrieved and combined data from five CSF datasets for discovery and two additional datasets for validation in 368 MS patients and controls. After data preprocessing, we calculated Z-scores for all datasets and for the integrated dataset. We used logistic regression models using training and validation datasets. We identified 11 differentially expressed proteins in the integrated dataset, revealing significant alterations in key pathways involved in immune response, neuroinflammation, and synaptic function. Notably, IGKC exhibited strong diagnostic potential, with an AUROC of 0.81. These findings highlight the value of re-analysing publicly available proteomics data to develop robust biomarker panels for MS diagnosis.

## 1. Introduction

Multiple sclerosis (MS) is a chronic and multifaceted neurodegenerative disease characterised by a wide range of neurological symptoms, including motor dysfunction, cognitive decline, and sensory disturbances [1]. Immune cell infiltration in the central nervous system (CNS) is the hallmark of MS, leading to local inflammation and demyelination of neurons [2,3]. MS is a progressive disease with appearance of symptoms at late stages of the disease. Therefore, identification of novel biomarkers for early diagnosis of MS will help clinicians to apply appropriate interventions, leading to improved quality of life for patients. Furthermore, early diagnosis of MS via protein biomarkers leads to decreased financial burdens on health organisations.

Despite frequent studies on the underlying mechanisms of MS and the identification of prognostic markers, many questions remain unanswered [4]. The variability in proteomic findings across studies complicates the identification of consistent biomarkers [5,6]. It impedes the development of reliable diagnostic tools for MS. This complexity underscores the critical need for reliable biomarkers to aid in diagnosis, prognosis, and therapeutic monitoring. In recent years, researchers have focused on the characterisation of protein and RNA biomarkers of MS in cerebrospinal fluid (CSF) [7].

Due to its close interaction with the CNS, CSF is a valuable source for identifying biomarkers in MS [8,9,10]. The influx of leukocytes from serum to CSF is highly filtered in healthy subjects [11]. Accordingly, CSF specimens are used to examine protein content and the number of cells. In this regard, McDonald’s criteria were developed and revised in 2017 to detect dissemination via CSF-specific IgG oligoclonal bands [12].

Proteomic studies have identified proteins potentially linked to MS in CSF [13,14,15,16]. However, only some of these findings have been consistently validated across independent cohorts. Reproducibility remains a crucial challenge, driven by small sample sizes, inconsistent diagnostic criteria, and variability in sample processing, data acquisition, and analysis. These factors contribute to the difficulty in establishing robust biomarkers for MS. Liquid chromatography–mass spectrometry (LC-MS)-based approaches are mainly used in this context to measure metabolites, including amino acids and proteins in body fluids [17].

Meta-analyses are powerful tools that overcome limitations in statistical power and reproducibility, and are used across biomedical research fields. Yet, their application in proteomics needs to be utilised more, especially where established biomarkers are lacking. Existing proteomics meta-analyses often rely on published results without accounting for the heterogeneity introduced by different databases and analytical strategies. We hypothesised that performing an unbiased meta-analysis of proteomics datasets could reveal a reliable set of MS-associated proteins with higher reproducibility and clinical relevance. To this end, we identified MS-focused CSF proteomic LC-MS datasets and re-analysed them using the analytical approach introduced by van Zalm and colleagues [18]. Using this approach, we identified biomarkers that were differentially expressed in CSF samples of MS subjects compared to the control subjects. To this end, we analysed datasets separately and then as one integrated sample. Enrichment analysis revealed related signalling pathways and processes. Then, we developed a model to evaluate the association between identified proteins and MS prevalence. To effectively mitigate the risk of overfitting in our trained model, we strategically employed a logistic regression model that not only validated our innovative two-pronged approach but also leveraged two recently published additional cohorts, which underscored the potential impact of our research.

## 2. Methods

### 2.1. Literature Review

A comprehensive search was conducted in the PubMed database to find MS-related CSF datasets. PubMed was prioritised since it is a comprehensive database and covers all studies including MS proteomics, which are supplemented by PRIDE (PRoteomics IDEntifications Database) for raw data. For this purpose, a combination of keywords was used: “MS” and “multiple sclerosis” as the first group, “proteomics”, “proteome”, and “protein” as the second group, and “CSF” or “cerebrospinal fluid” as the third groups of keywords in different combinations. Due to the limited available datasets, brain and spinal cord analyses were not included in this study.

The inclusion criteria included proteomic analyses involving the brain, the spinal cord, CSF, and other relevant tissues. This applied to MS patients and control subjects, with studies focusing on various MS subtypes versus controls. Additionally, the proteomic profiling utilised liquid LC-MS/MS in data-dependent acquisition (DDA) mode and required high-resolution and high-accuracy instrumentation. Exclusion criteria included review studies, studies with no raw data from LC-MS (proteomics), studies written in languages other than English, studies with non-human samples, studies with no MS-related samples, studies with no description of sample preparation and mass spec techniques, and for proteomics, the use of non-DDA methods (e.g., SRM, MRM, PRM, Western Blot, 2D gel electrophoresis).

The included articles assessed data availability in the databases using PRIDE and ProteomeXchange (https://proteomecentral.proteomexchange.org, accessed on 5 August 2024). Data were retrieved from the available datasets and used for further analysis. Additionally, we searched for all proteomics datasets available for MS patients in the same databases. Ultimately, we included eligible datasets with available proteome datasets (*n* = 6) and 1 dataset with no available published articles from the PRIDE database.

The present systematic review and meta-analysis included all the datasets with corresponding papers and PubMed identifiers. The PubMed database was accessed online through the National Centre for Biotechnology Information (NCBI) website (https://pubmed.ncbi.nlm.nih.gov/, accessed on 3 August 2024). One unpublished dataset in the PRIDE database was added to the datasets in this study.

### 2.2. Proteomics Analysis

All raw LC-MS data were retrieved and analysed using FragPipe 22.0 via *Homo sapiens* protein sequences from the UniProtKB/Swiss-prot database, which included 40,936 proteins in total and was downloaded on 10 July 2024. The analysis settings for amino acids included a maximum of two missed tryptic cleavages and a peptide length between 7 and 50 amino acids long. In addition, the cysteine residues were set up as fixed. In contrast, acetylation of the N-terminal of proteins and methionine oxidation were set up as variable modifications, and the maximum number of modifications was three. Modification on the N-terminal of the peptides and lysine residues in tandem mass tag (TMT) studies was used as a fixed modification. A 1% false discovery rate (FDR) was applied for both Percolator and ProteinProphet [19]. For IonQuant [20], the match between runs was set up for all the positions where at least one ion for peptide quantification was needed.

### 2.3. Data Analysis

Protein identification data were imported into RStudio (v.4.4.2) for preprocessing, statistical analysis, and visualisation. The source code for this study is publicly available at the following GitHub Repository: https://github.com/ElnazAmanzadeh/MS-Proteome-meta-analysis, accessed on 5 August 2024.

All datasets were normalised in R (v.4.4.2), while two distinct methods were applied based on the presence or absence of reference nodes in datasets. Determining the normalisation factor (NF) was crucial to calculating standardised intensities for all samples. For this purpose, the sum of intensities was calculated for each sample in all datasets. In addition, the median of all summed intensities was calculated for each dataset separately. Then, the normalisation factors were calculated for each dataset sample via division of the median of summed intensities by the summed intensity of each dataset sample. In all datasets with reference samples, the median intensities were calculated and then used to calculate the NF. On the other hand, in studies with several reference samples, the NF was determined via calculation of the median intensity of the reference samples. In the following, this factor was recruited to normalise the protein intensities of each sample.

In the present study, we used the method by Zalm and colleagues [18]. This method does not rely on principal component analysis (PCA) to identify and remove outliers but rather uses a standardised outlier identification approach to optimise the results. However, a PCA plot was used to visualise the results before and after identifying and removing outliers. Next, normalised datasets were used to create a theoretical sample based on each dataset’s median intensities achieved for individual proteins. Then, the correlation between each sample and the theoretical median sample was calculated using the Pearson correlation test. Additionally, the correlation coefficients were used to calculate the standard deviations. Simultaneously, outliers were defined as values beyond three standard deviations, which were subsequently removed.

In this present study, we encountered various methodologies for sample preparation from the CSF, experiment set-ups, and profiling of the proteins. Therefore, CSF samples were adjusted primarily to remove batch effects. We utilised data from various datasets to calculate Z-scores to achieve this goal, enabling effective comparisons and deeper insights. To calculate the Z-score, a log2 transformation was applied to the intensities of all samples. Then, the mean and standard deviation were calculated for a protein in different samples using the intensities of control samples. Next, the Z-score of each protein in all samples was calculated, and this was repeated for all proteins across all samples. Ultimately, all datasets were merged into a large dataset for further analysis.

MS and control samples were identified using the information in metadata including demographic data and clinical criteria. 

To accomplish this, we used RStudio to perform statistical analyses. Comparison of MS and control samples for identification of proteins with statistically significant expression was performed using Fisher’s exact test. Those with less than 70% expression in samples were removed to filter out low-frequency proteins. Next, a non-parametric Mann–Whitney U test was used for statistical analysis because parametric tests did not apply to these data. In addition, the Benjamini–Hochberg procedure was used to correct the obtained *p*-values. Importantly, the results of the quantitative expression analysis for the proteins in each dataset were obtained to be compared with the meta-analysis results. For this, we used Student’s *t*-test and Benjamini–Hochberg correction.

During the meta-analysis, functional enrichment analysis was performed to find the signalling pathways, including the identified significantly expressed proteins. To accomplish this, the Clusterprofiler package for R was used to visualise significant signalling pathways using MSidDB Gene Sets Hallmark datasets via misgdbr-package. Additionally, Metascape pathway analysis [21] and EnrichR [22,23] were employed for comprehensive Gene Ontology (GO) analysis, focusing on biological processes and molecular functions. These combined approaches provided an in-depth understanding of the biological significance and functional roles of the identified proteins.

EnrichR generated ranked lists of enriched terms for each selected gene set library, using both adjusted *p*-values (e.g., Benjamini–Hochberg correction) and integrated combined scores (which incorporate *p*-values and z-scores) to prioritise biologically and clinically relevant findings. This statistical rigour ensured that top-ranked terms reflected the most reliable and meaningful associations with the observed proteomic alterations in Parkinson’s Disease.

### 2.4. Model Development and Validation

Datasets from Timirci (2019) [24] and Comabella (2021) [25] were used as validation datasets for CSF samples. Tabular quantification datasets were used for primary model development using data for proteins identified as biomarkers. Then, a logistic regression model was applied to the integrated biomarker panel. Logistic regression was selected due to its interpretability and examinability. The training models were developed using four datasets for CSF samples. Then, to test the validation of the model, two independent models were created using the datasets mentioned before. The identified markers and developed models were tested on validation datasets to ensure no overfitting. The pROC R package was used to test the models. The results were evaluated using area under the curve plots in a receiver operating characteristic (ROC) curve analysis. Then, the area under the receiver operating characteristic curve (AUROC) was calculated for each protein to evaluate the efficacy of biomarker candidates identified through the meta-analysis. Finally, using the brute-force method, a collection of proteins from CSF samples was tested in 3-protein combinations in two validation datasets.

## 3. Results

### 3.1. Study Selection

The search process was based on Preferred Reporting Items for Systematic Reviews and Meta-Analyses (PRISMA) guidelines. The search process led to the primary finding of 493 papers. Screening of titles led to the exclusion of 142 duplicate papers. Article-type screening resulted in the exclusion of 48 review articles. Abstracts and full-text articles were screened to find the documents that had no raw data available, used non-human samples, or used cell lines. This stage of screening led to the exclusion of 280 articles. Screening of studies with no available raw data, non-English-language studies, and studies using non-human samples excluded 4, 14, and 5 papers, respectively.

**Figure 1:**
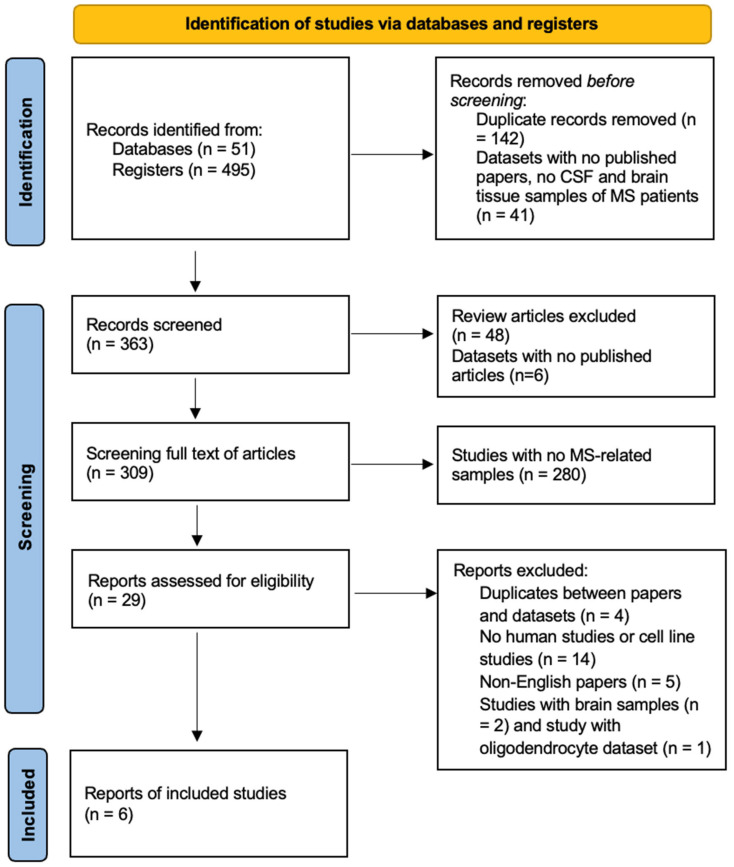
PRISMA flow diagram of search and study selection process.

Meanwhile, from eight datasets with available raw data, four included data from non-MS diseases and MS patients treated with therapeutic compounds and methods. Finally, four eligible datasets were included in the present study. In addition, searching for MS datasets in databases yielded 51 datasets. However, 41 datasets were excluded since they included cell line studies, extracellular vesicle (EV) samples, treated MS samples, and blood and plasma samples. Further, the search yielded two studies with brain and one dataset with oligodendrocyte proteomics data, which were excluded since the meta-analysis needed four or more studies to complete the meta-analysis and validation processes. In total, seven studies that used CSF samples for proteomic analysis were included in this systematic review and meta-analysis. The study selection process is represented in Figure 1.

### 3.2. Data Extraction

Table 1 represents the characteristics of the included studies and related proteomics datasets that used CSF and brain samples from MS patients.

### 3.3. Data Preprocessing

**Figure 2:**
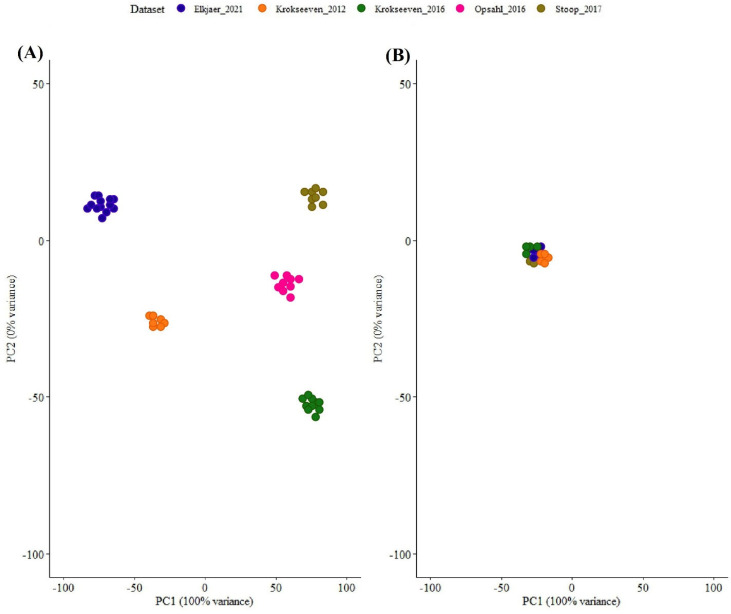
Z−score transformation for adjustment of data and removal of batch effects. (**A**) Pearson correlation was calculated between each sample, and theoretical samples more than three standard deviations removed from the samples were considered as outliers and removed from downstream analysis. This process was repeated for each sample, resulting in a PCA plot. (**B**) Z−score transformation was applied to overcome variations between datasets, resulting in a homogeneous dataset. The datasets used in this analysis included Elkjaer et al., 2021 [7]; Krokseveen et al., 2012 [28]; Krokseveen et al., 2016 [29]; Opsahl et al., 2016 [27]; and Stoop et al., 2017 [26].

LC-MS data for each study were retrieved from the PRIDE dataset using relevant dataset IDs in the articles or from support data published with the articles. For the studies with available raw data, these data were searched against the UniProt Human protein canonical sequence database (downloaded on 20 July 2024: 40,936 entries) using MSFragger/Fragpipe. This approach minimised variability due to the protein sequence differences, searching algorithm, quantification, and samples. In addition, using a canonical protein database led to removing issues, including spurious peptide spectral matches and artificial isoforms. Data normalisation was performed using the median intensities of reference samples. The median of summed intensities of samples was used as the NF for samples with no reference node. Outliers in samples were identified using a method developed by Zalm and colleagues (2023) [18]. In this method, protein expression levels should have correlations between different samples; if this is not observed, it represents a problem in the experimental stages. Therefore, the intensities of proteins in each sample are correlated with the median intensity of all samples per dataset. Outlier proteins were identified using a threshold of three standard deviations of mean correlation for each dataset. Six samples were removed since they were identified as outliers in CSF samples (Figure S1A). In brain samples, no sample was identified as an outlier (Figure S1B). As Zalm and colleagues [18] have mentioned, the model they developed led to fast and accurate data adjustment, helping to improve downstream analyses. In addition, Z-scores were calculated for each dataset individually and then for the integrated dataset, and then they were used for the normalisation of protein intensities. This approach helped remove batch effects caused by differences in experimental steps, sample preparation, and measurement instruments. PCA plots of datasets before and after normalisation via Z-score for both CSF samples are represented in Figure 2.

### 3.4. Discovery of Biomarker Candidates

**Figure 3:**
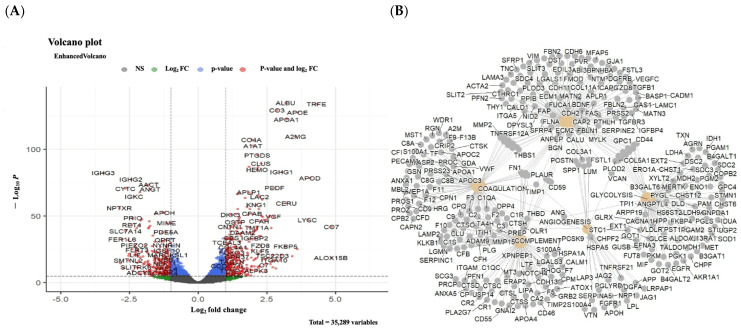
Statistical analysis and enrichment analysis results: (**A**) The results of the Mann–Whitney U test and Benjamini–Hochberg comparison correction, resulting in the identification of 73 biomarkers for CSF samples. Grey: proteins expression levels that are not significantly changes, red: proteins which significantly upregulated, blue: proteins which significantly upregulated, green: proteins with upregulation which don’t meet the fold change cutoffs. (**B**) Biomarker candidates in CSF samples were used to perform enrichment analysis using the MSigDB Hallmark database for CSF samples.

After removing outliers in the CSF group, the remaining samples included 127,083 proteins. Then, Fisher’s exact test was performed to find the proteins with expression in only MS or control cases. However, this test did not yield to a significant number of proteins using a *p*-value < 0.05 as a substantial cut-off value (Figure S2A,B). Therefore, we decided to keep all the proteins in the further analysis steps. In the following steps, the non-parametric Mann–Whitney U test and then Benjamini–Hochberg multiple testing were used for correction. The results showed 73 proteins in CSF samples (Figure 3A) with a statistically significant increase in expression levels which were used for identification of functional interactions between potential biomarkers and enrichment analysis using the Molecular Signatures Database (MSigDB) Hallmark dataset with the highest statistical confidence level for both CSF and brain samples (Figure 3B). This analysis emphasises the importance of the effect of protein networks and metabolism in MS. Surprisingly, some of the proteins found to be significantly expressed in the meta-analysis did not show significant increases or decreases in expression levels in individual sample analysis. This finding indicates that meta-analysis helps to discover novel biomarkers not previously identified.

**Figure 4:**
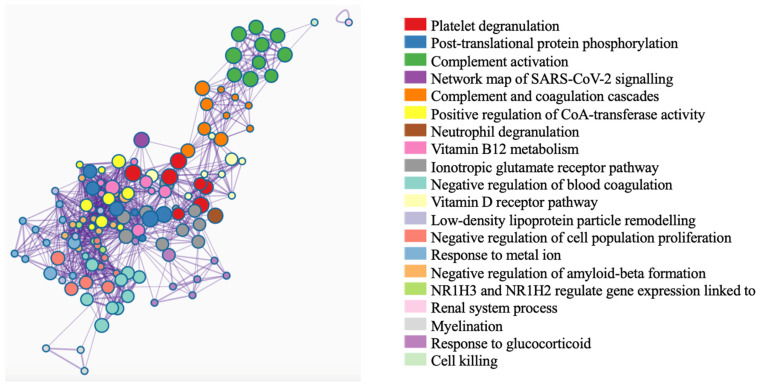
Network of enriched terms: This network visualisation shows enriched biological terms, with nodes coloured by cluster ID to indicate functional groupings. Terms within the same cluster are positioned closer together, reflecting higher functional similarity. Node size represents the number of genes associated with each term.

The network of enriched biological terms is shown in Figure 4, emphasising key functional clusters and their interconnections, which provide crucial insights into the molecular mechanisms of the disease. Functionally related terms are grouped into distinct clusters, such as “Platelet Degranulation”, “Complement Activation”, and “Vitamin D Receptor Pathway”, underscoring the interplay between immune regulation, coagulation, and metabolic processes. Terms like “Platelet Degranulation” and “Post-Translational Protein Phosphorylation” stand out for their strong statistical enrichment and extensive gene representation, highlighting their critical roles in processes such as cellular signalling, protein modification, and immune activation. The use of colour to represent statistical significance further emphasises the pathways most relevant to the disease, with darker nodes indicating higher significance. This network not only illustrates the complexity of the biological interactions but also identifies priority pathways that could serve as potential therapeutic targets or provide deeper insights into the disease’s progression and pathology.

**Figure 5:**
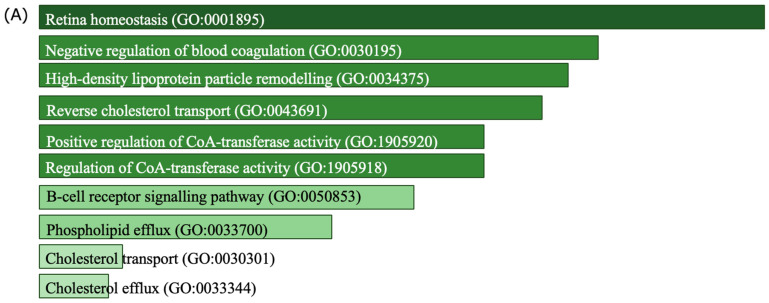

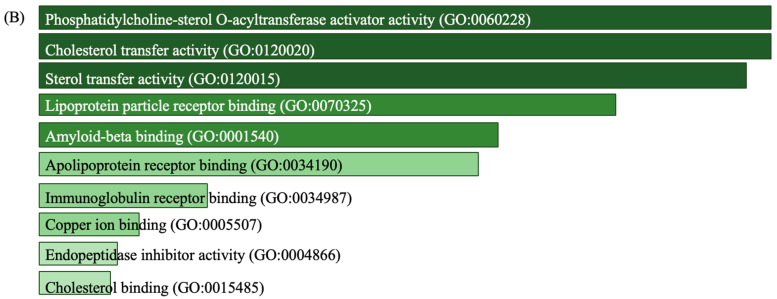
Enriched GO terms for biological processes and molecular functions (2023). The bar charts display the most significantly enriched (**A**) biological processes and (**B**) molecular functions identified through GO analysis. Longer bars indicate higher levels of enrichment. GO terms and their identifiers are provided for each process and function, highlighting pathways and molecular activities relevant to this study. Biological processes are shown in (**A**), while molecular functions are shown in (**B**).

Figure 5 highlights the most significantly enriched GO terms, providing insight into the biological processes (Figure 5A) and molecular functions (Figure 5B) associated with the differentially expressed proteins identified in this meta-analysis. In the biological processes category, pathways such as “Retina Homeostasis” (GO:0001895) and “Negative Regulation of Blood Coagulation” (GO:0030195) were highly enriched, indicating their potential relevance to maintaining tissue integrity and regulating pathological responses. Other enriched terms, including “High-Density Lipoprotein Particle Remodelling” (GO:0034375) and “Reverse Cholesterol Transport” (GO:0043691), suggest critical roles in lipid metabolism and transport, processes known to be implicated in neurodegenerative and systemic diseases [30,31].

Similarly, the molecular functions analysis revealed significant enrichment for activities such as “Cholesterol Transfer Activity” (GO:0120020) and “Amyloid-Beta Binding” (GO:0001540). These findings suggest involvement in cholesterol transport and amyloid protein interactions, both of which are crucial in cellular signalling and potentially linked to disease pathophysiology. Notably, “Lipoprotein Particle Receptor Binding” (GO:0070325) and “Phosphatidylcholine-Sterol O-acyltransferase Activator Activity” (GO:0060228) further highlight the relevance of lipid metabolism and receptor-mediated interactions in the context of MS. These enriched terms collectively provide evidence of the intricate involvement of lipid regulation, protein interactions, and homeostasis in the biological mechanisms underlying the studied condition.

**Figure 6:**
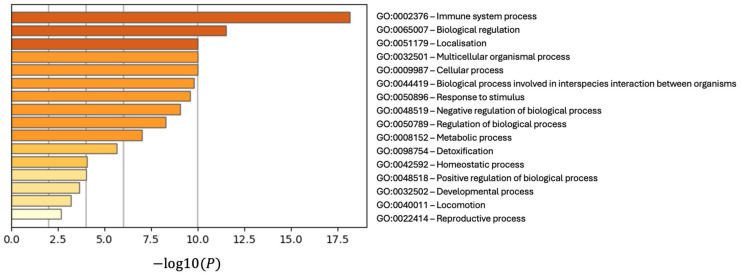
The bar chart shows the top 20 enriched GO terms (1 per cluster) ranked by −log10(*p*-value), with higher bars indicating greater significance. The count represents the number of input genes associated with each term, while the percentage (%) reflects the proportion of genes linked to the term. Multi-test adjusted *p*-values (−log10(q)) ensure statistical robustness.

The GO enrichment analysis of differentially expressed proteins (DEPs) identified significant associations with key biological processes (Figure 6), with immune system processes (GO:0002376) showing the highest enrichment (*p* < 10−17), emphasising the pivotal role of immune dysregulation in the pathology of the disease. This highlights the involvement of DEPs in immune-related pathways, consistent with the inflammatory mechanisms commonly observed in the condition. The second most significant process, biological regulation (GO:0065007), reflects the role of DEPs in critical regulatory networks, including the modulation of cellular responses and protein activity, suggesting that disruptions in these pathways may drive disease progression. The third, localisation (GO:0051179), underscores the involvement of DEPs in intracellular and intercellular transport processes essential for cellular function, with potential implications for molecular trafficking disruptions in the disease. Collectively, these findings provide critical insights into the immune, regulatory, and transport-related roles of the identified DEPs, offering a foundation for understanding the disease’s molecular underpinnings and identifying therapeutic targets.

### 3.5. Development of a Model Using Biomarker Candidates

**Figure 7:**
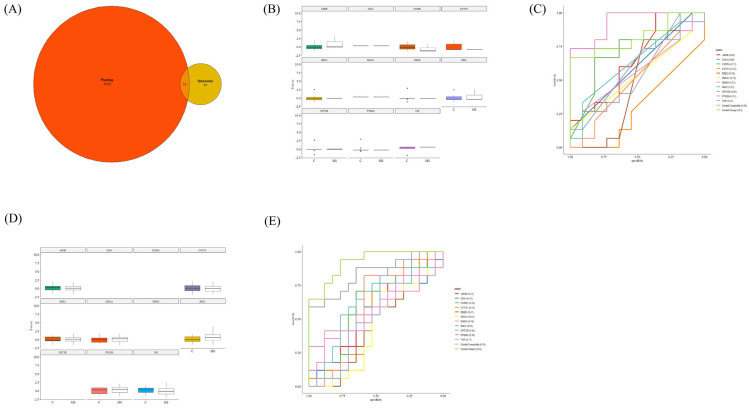
Biomarker validation cohorts. (**A**) Venn diagram showing 11 overlapping proteins (Log2FoldChange) between the test and pruning cohorts, including APOE (3.614), CD14 (−2.050), CNDP1 (−1.548), CTN1 (1.379), DKK3 (1.183), IGHA1 (1.937), IGHG3 (−3.451), IGKC (−2.326), NPTXR (−2.852), PTGDS (2.162), and VGF (2.747). (**B**,**D**) Post-analysis of two datasets, including Comabella et al. [25] and Timirci et al. [24], was used to evaluate the biomarker efficacy of these 11 biomarker candidates. Each of the 11 proteins revealed significant differences between MS and controls in both datasets. (**C**,**E**) Further validation analysis was performed using a logistic regression model for 11 biomarker candidates, which was trained and tested using both models on the two validation cohorts (Comabella et al. [25] and Timirci et al. [24]). AUROCs for individual proteins as well as the two models, are shown in the legend.

In further analysis, putative biomarkers of CSF samples were used to develop machine-learning models using logistic regression. Considering that in-house samples for the validation analysis were not available, we used two datasets for the two-step validation of our models. To this end, we used five CSF datasets for primary model development and two datasets, including Timirci (2019) [24] and Comabella (2021) [25], for model validation. All the steps were performed for model validation datasets, including pre-processing, Z-score calculation, normalisation, and exploratory analysis. Meanwhile, the most effective proteins shared between test datasets and validation datasets on model accuracy were also found for each dataset. APOE, CD14, CTNPD1, CTNT1, DKK3, IHGA1, IGHG3, IGKC, NPTXR, PTGDS, and VGF were found for CSF samples, among which the highest AUROC was achieved for IGKC as 0.81 (Figure 7A–C).

## 4. Discussion

In this meta-analysis of CSF proteomes from MS patients, we identified several proteins that may play important roles in MS pathogenesis. Proteins such as APOE, CD14, CNDP1, CTNT1, DKK3, IGHA1, IGHG3, IGKC, NPTXR, PTGDS, and VGF provide valuable insights into the complex and interrelated mechanisms of immune regulation, neurodegeneration, and cellular signalling in MS.

APOE, traditionally recognised for its involvement in lipid metabolism, is implicated as a crucial modulator of inflammation in the CNS [32]. Beyond its lipid-related functions, APOE may influence T-cell proliferation, macrophage activity, and antigen presentation by CD1 molecules to natural killer T (NKT) cells [33]. Its potential role in dampening neuroinflammation positions APOE as a candidate modulator of MS-related immune responses, potentially shaping the immune landscape during disease progression [34].

CD14, a co-receptor for lipopolysaccharides, is another key protein identified in our analysis. Its upregulation in microglia and macrophages during active disease phases contributes to the production of proinflammatory cytokines, such as TNF-α and IL-1β, which drive demyelination and neurodegeneration [35,36]. Elevated CD14 levels in progressive MS forms highlight its association with disease severity [37,38], suggesting its potential as a marker of neuroinflammation and therapeutic targeting.

While no direct interaction between APOE and CD14 is confirmed in MS, both proteins modulate innate immune responses and may contribute to a shared inflammatory axis worth further investigation.

Immunoglobulins such as IGHA1, IGHG3, and IGKC were also identified as DEPs in the CSF of MS patients. These proteins are central to adaptive immune responses and have long been linked to MS pathology. The presence of oligoclonal bands (OCBs) in the CSF, indicative of intrathecal immunoglobulin production by B-cell clones, further supports the role of B-cell dysregulation in MS. Elevated levels of IGHG3 and IGKC, components of IgG molecules, suggest ongoing humoral immune responses in the CNS, aiding in the distinction of MS from other neurological disorders [39,40]. The identification of IGHA1, an immunoglobulin A heavy chain, is particularly intriguing, as it may reflect broader immune activity, despite its direct role in MS pathology remaining unclear.

Similarly, reductions in CNDP1 (carnosine dipeptidase 1) support its proposed protective role in MS pathology. As an enzyme that breaks down the neuroprotective dipeptide carnosine, CNDP1 helps buffer oxidative stress—a factor elevated in MS [41,42]. This protein’s function in reducing oxidative damage may also promote oligodendrocyte survival, linking it to remyelination and its relevance in relapsing–remitting MS (RRMS), where oxidative stress exacerbates disease relapses.

DKK3 (Dickkopf-3) was identified for its involvement in modulating Wnt/β-catenin signalling pathways and immune responses [43]. Emerging evidence suggests that DKK3 influences local T-cell polarisation and cytokine production, central to MS pathogenesis [44,45]. Its potential role in modulating immune tolerance within the CNS could open new avenues for therapeutic modulation of autoimmunity in MS.

NPTXR (neuronal pentraxin receptor) is another protein linked to neurodegenerative processes [46]. Its decreased levels in the CSF of MS patients, particularly in those with progressive disease, highlight its role in maintaining synaptic integrity [28,41,46]. Synaptic dysfunction, a hallmark of progressive MS, contributes to cognitive and motor decline, and NPTXR’s association with synaptic health suggests it could serve as a marker for neurodegeneration in MS.

PTGDS (prostaglandin D2 synthase) is highly expressed in oligodendrocytes and astrocytes, implicating it in remyelination and inflammatory modulation [47,48]. PTGDS exhibits both neuroprotective and proinflammatory activity, depending on disease stage [48,49,50], underscoring its complex involvement in MS pathophysiology.

VGF, a neuropeptide involved in synaptic plasticity and neuroprotection, was also significantly upregulated in MS patients. Its potential role in balancing excitatory and inhibitory signals in neurons makes it a key contributor to the neurodegenerative processes seen in MS, particularly cognitive decline [51,52]. VGF’s regulation by inflammatory signals further supports its involvement in neuroinflammation and neurodegeneration in MS.

Collectively, these proteins and their interactions underscore the potential for developing personalised therapeutic strategies for MS. Targeting the APOE-CD14 axis or modulating immunoglobulin production could provide tailored treatments for patients exhibiting particular immune profiles.

Beyond these individual proteins, integrative pathway enrichment analyses offer a broader, systems-level perspective. Clustering of terms such as “Platelet Degranulation,” “Complement Activation,” and “Vitamin D Receptor Pathway” highlights the complex interplay between immune responses, coagulation, and metabolic regulation in MS, and highlights the value of meta-analyses in the context of heterogeneous disease. The prominence of “Post-Translational Protein Phosphorylation” further points to intricate protein modifications orchestrating disease-associated signalling. 

The enrichment of immune system processes among DEPs solidifies the centrality of immune dysregulation in MS. Additionally, dysregulations in biological regulation, intracellular transport, and regulatory networks may impede cellular homeostasis and molecular trafficking, contributing to disease progression. Processes like “Retina Homeostasis” and “Negative Regulation of Blood Coagulation” highlight the potential for both localised and systemic dysregulation, while molecular functions involving “Cholesterol Transfer Activity” and “Amyloid-Beta Binding” underscore the significance of lipid metabolism and protein aggregation in MS pathology.

In this study, we identified several pathways and biological processes that align with findings from our previous results [53]. Specifically, the complement and coagulation cascades emerged as a shared pathway, consistent with their central role in both immune response and neuroinflammation. Similarly, processes related to cholesterol metabolism, such as high-density lipoprotein particle remodelling, cholesterol efflux, and reverse cholesterol transport, align with previously reported pathways like the vitamin digestion and absorption pathway. Furthermore, amyloid-beta binding identified in this study complements the prion disease pathway observed in earlier findings, highlighting common mechanisms involving protein misfolding and aggregation. Finally, immune system-related processes, including complement activation and the negative regulation of biological processes, mirror pathways such as the NOD-like receptor signalling pathway reported in our prior work [53]. 

In conclusion, this meta-analysis highlights several CSF proteins and pathways of importance in MS pathogenesis, particularly in CSF. The identified proteins—such as APOE, CD14, CNDP1, DKK3, NPTXR, PTGDS, and VGF—underscore the multifaceted nature of MS, encompassing immune dysregulation, neuroinflammation, and neurodegeneration. Furthermore, the elevated levels of immunoglobulins, including IGHA1, IGHG3, and IGKC, reinforce the importance of humoral immune responses in MS. Understanding the diverse roles these proteins play in MS not only deepens our knowledge of the disease mechanisms but also offers new potential targets for therapeutic intervention. Future research focused on the functional roles of these proteins in disease progression will be essential for advancing MS treatments.

## 5. Conclusions

This study presents a comprehensive meta-analysis of proteomics data from MS patients’ CSF, resulting in the identification of several essential proteins, including APOE, CD14, and PTGDS for neuroinflammation, CNDP1, NPTXR, and VGF for neurodegeneration, CTNT1 and DKK3 for repair mechanisms, and IGHA1, IGHG3, and IGKC as hallmarks of MS. By leveraging independent datasets, we provide more reliable biomarkers highlighting immune dysregulation, neuroinflammation, and neurodegeneration as central mechanisms in MS pathogenesis.

Our findings underscore the utility of combining proteomics data from diverse sources to improve the robustness of biomarker discovery. Future studies should focus on validating these proteins across different MS subtypes and other neurological conditions to establish their clinical relevance further. In addition, further prognostic and diagnostic models should be developed for early diagnosis of MS appearance and progression.

In moving beyond existing research, our findings demonstrate the power of integrating proteomics data from diverse sources to enhance the robustness of biomarker discovery. These proteins have the potential to be incorporated into diagnostic panels, allowing for earlier and more accurate detection of MS. Moreover, by identifying proteins linked to distinct pathological mechanisms, our study lays the groundwork for stratifying MS patients based on their molecular profiles, opening up possibilities for personalised treatment approaches, while improving understanding of pathophysiology. Future studies should focus on validating these proteins across different MS subtypes and other neurological conditions to further validate clinical applicability.

## 6. Limitations

While this meta-analysis provides valuable insights into MS pathogenesis, it has several limitations. First, the datasets included were heterogeneous, with variability in sample preparation, data processing, proteomic platforms and patient characteristics, particularly regarding MS subtypes, disease stages and control group definitions. Such inconsistencies introduce technical and biological variability that may affect protein quantification and reproducibility. Although stringent normalisation and outlier removal procedures were applied, residual batch effects and methodological differences may still influence the results.

Secondly, the limited availability of detailed metadata, such as patient demographics, treatment history, and control group diagnoses, restricted the ability to conduct detailed subgroup analyses and may have masked protein expression patterns specific to disease stages or clinical phenotypes. Thirdly, differences in control groups across studies (e.g., healthy individuals vs. patients with other neurological conditions) may confound the interpretation of differentially expressed proteins. Finally, while this study focuses on CSF, it does not account for potential proteomic changes in other compartments, such as blood. Further validation in larger, more diverse cohorts and across different biofluids will be necessary to confirm the clinical relevance of these findings.

## Figures and Tables

**Table 1:** Summary of the included papers and datasets.

Sample ID	Author, Year	Number of Control Samples	Number of MS Samples	Sample Type
5	Timirci, 2019 [24]	19	23	CSF
6	Stoop, 2017 [26]	45	47	CSF
7	Opsahl, 2016 [27]	14	50	CSF
8	Krokseeven, 2012 [28]	17	17	CSF
PXD004572	Kroksveen, 2017 [29]	21	21	CSF
PXD017643	Elkjaer, 2021 [7]	33	103	CSF
PXD022958	Comabella, 2021 [25]	44	28	CSF
PXD004540	Kroksveen, 2016 [29]	21	216	CSF

The studies with No ID represent datasets retrieved using published articles and support data unavailable via proteomics datasets.

## Supplementary Materials

The following supporting information can be downloaded at: https://www.mdpi.com/article/10.3390/ijms26136171/s1.
