# Supplementary material for: Comprehensive Meta-Analysis of Differentially Expressed Proteins in Cerebrospinal Fluid Associated with Multiple Sclerosis
Source: Int J Mol Sci. 2025 Jun 26;26(13):6171. doi: 10.3390/ijms26136171 (PMC12249574; doi:10.3390/ijms26136171)

Supplementary file : Comprehensive Meta-Analysis of Differentially Expressed Proteins in Cerebrospinal Fluid Associated with Multiple Sclerosis

Figure S1 A: PCA plots representing the outlier selection method. Blue dots represent outlier samples in all plots, and red indicates non-outlier samples. (A) Krokseeven, 2012, (B) Krokseeven, 2016, (C) Opsahl, 2016, (D) Stoop, 2017, (D) Timirci, 2019, (E) Elkjaer, 2021, (F) Comabella, 2021.

<table>
<tr>
<td>(A)
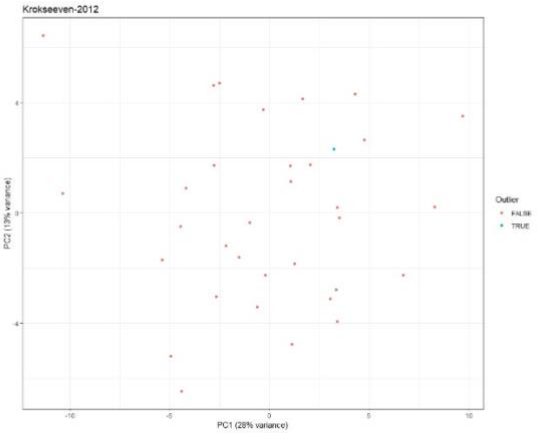
</td>
<td>(B)
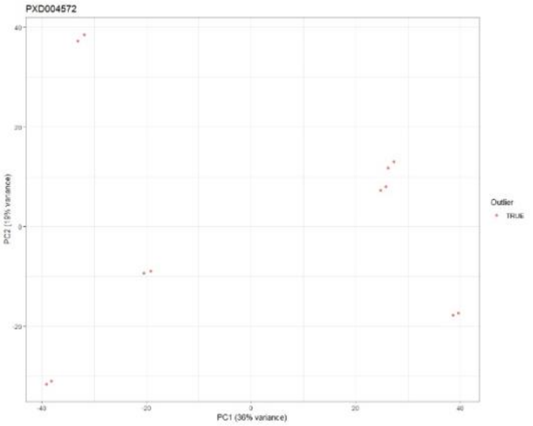
</td>
</tr>
<tr>
<td>(C)</td>
<td>(D)</td>
</tr>
</table>

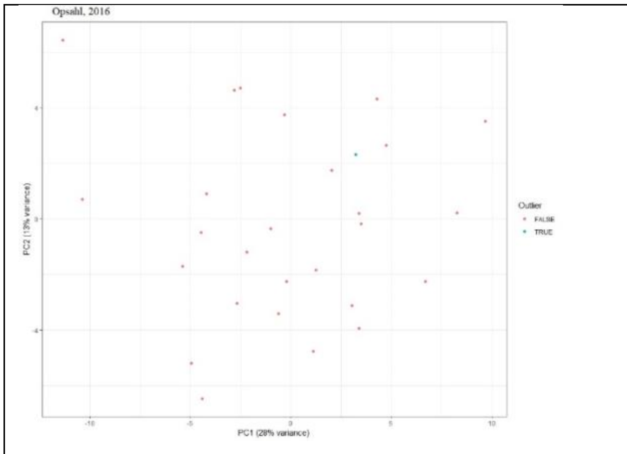

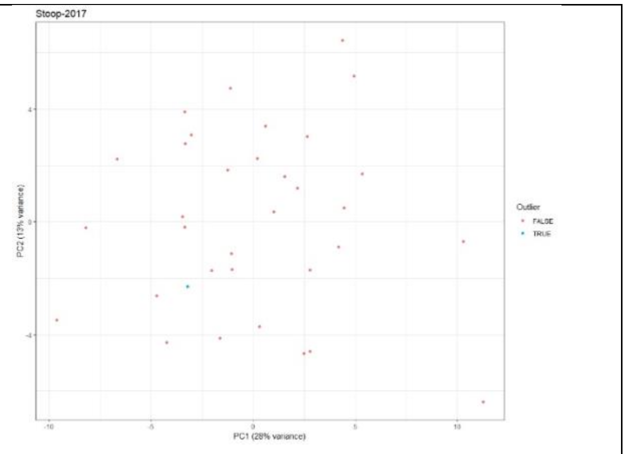

(E)

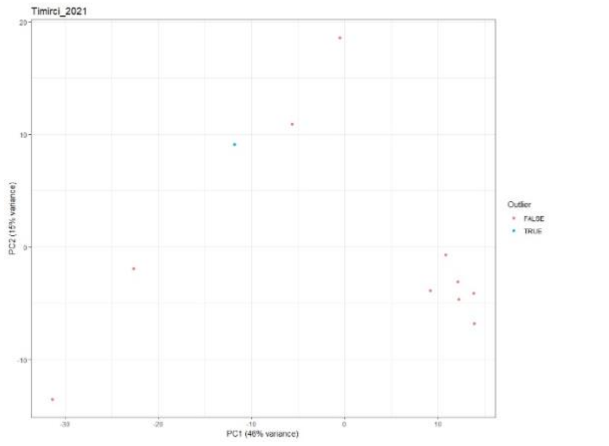

(F)

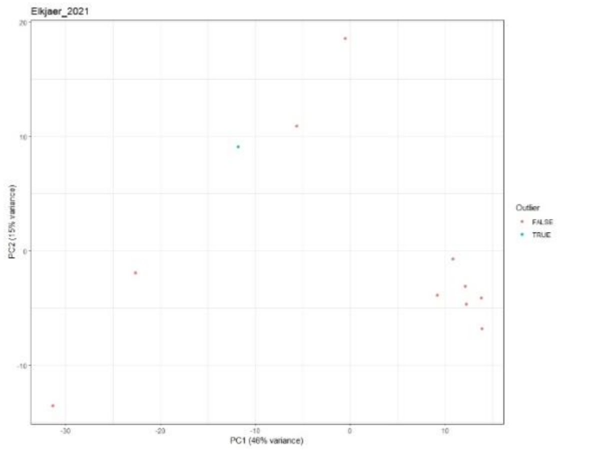

(F)

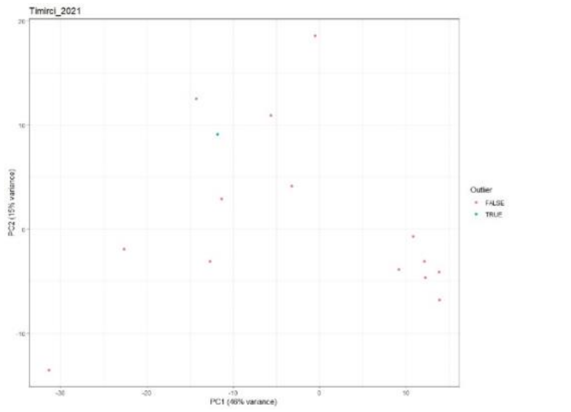

Figure S2: Heatmap representing the quantitative analysis results for proteins before and after filtering out during the discovery analysis. Heat maps of all proteins were drawn using Z-scores.

(A) CSF samples were used in this analysis. (B) Heatmap of CSF samples after applying the 70% cut-off.

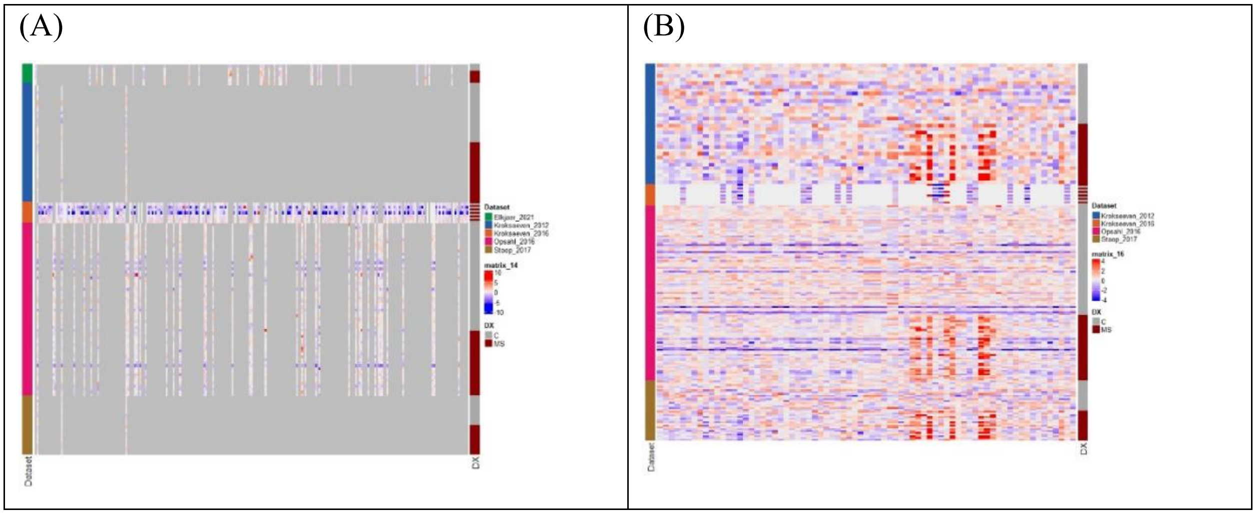

Supplement: Supplementary file 1 [file ijms-26-06171-s001.zip › Supplementary file.pdf]
