# Supplementary figures and images for: Comprehensive Meta-Analysis of Differentially Expressed Proteins in Cerebrospinal Fluid Associated with Multiple Sclerosis
Source: Int J Mol Sci. 2025 Jun 26;26(13):6171. doi: 10.3390/ijms26136171 (PMC12249574; doi:10.3390/ijms26136171)

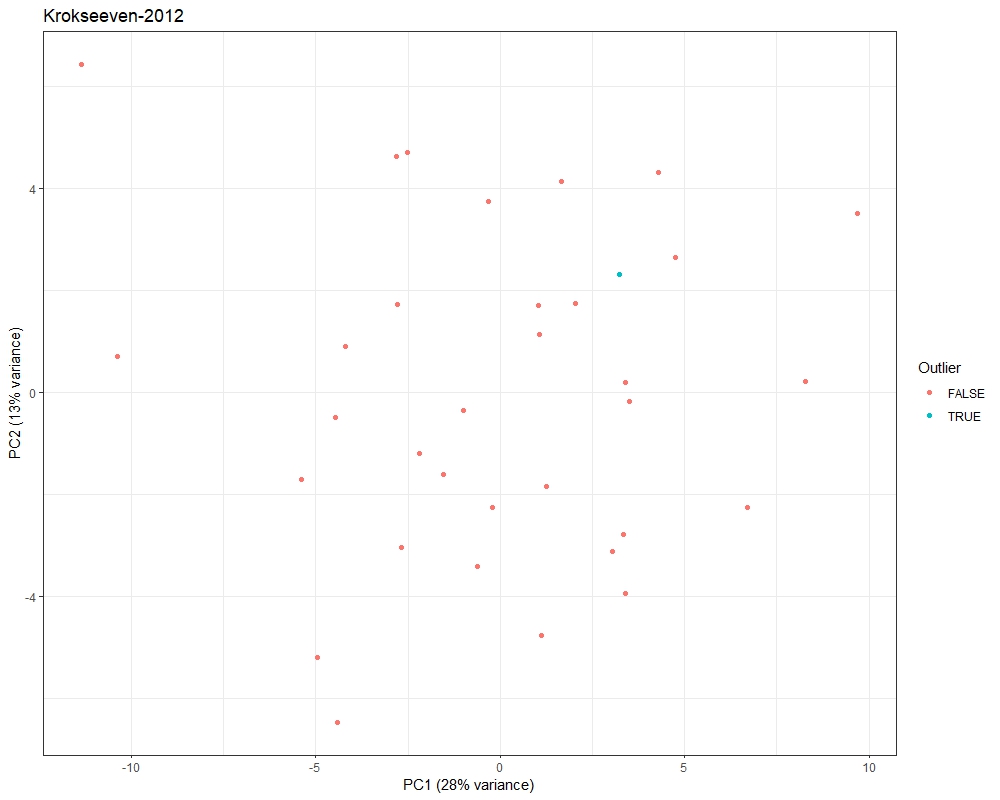

Supplement: Supplementary file 1 [file ijms-26-06171-s001.zip › Figure-S1-A.jpeg]

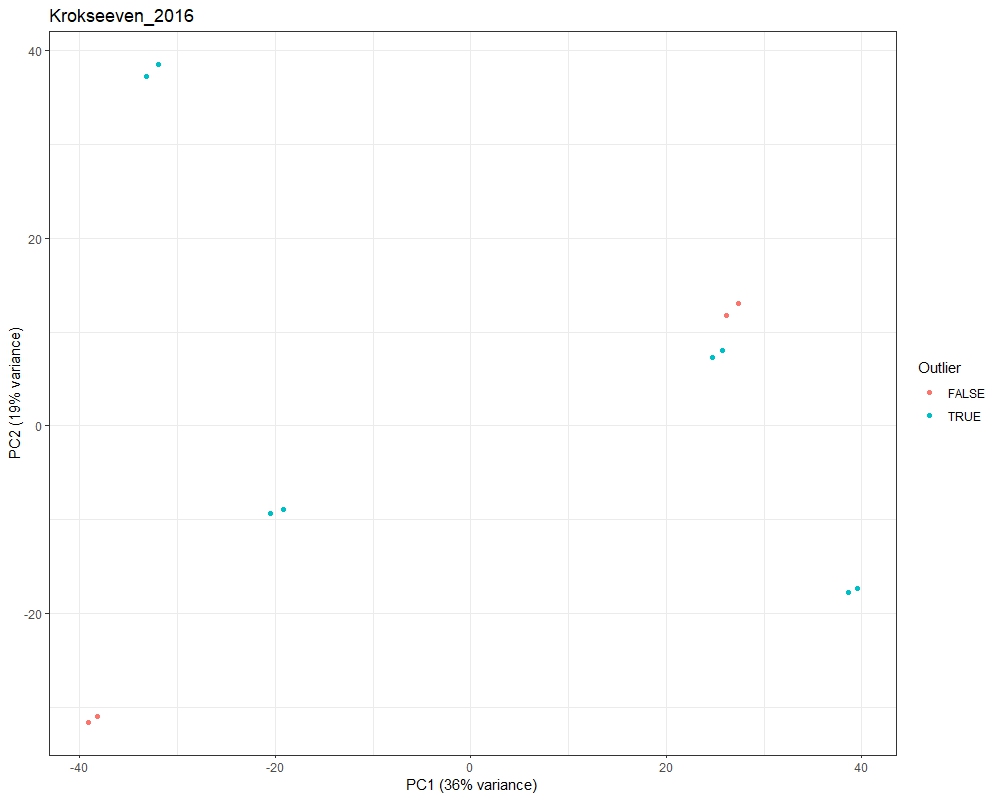

Supplement: Supplementary file 1 [file ijms-26-06171-s001.zip › Figure-S1-B.jpeg]

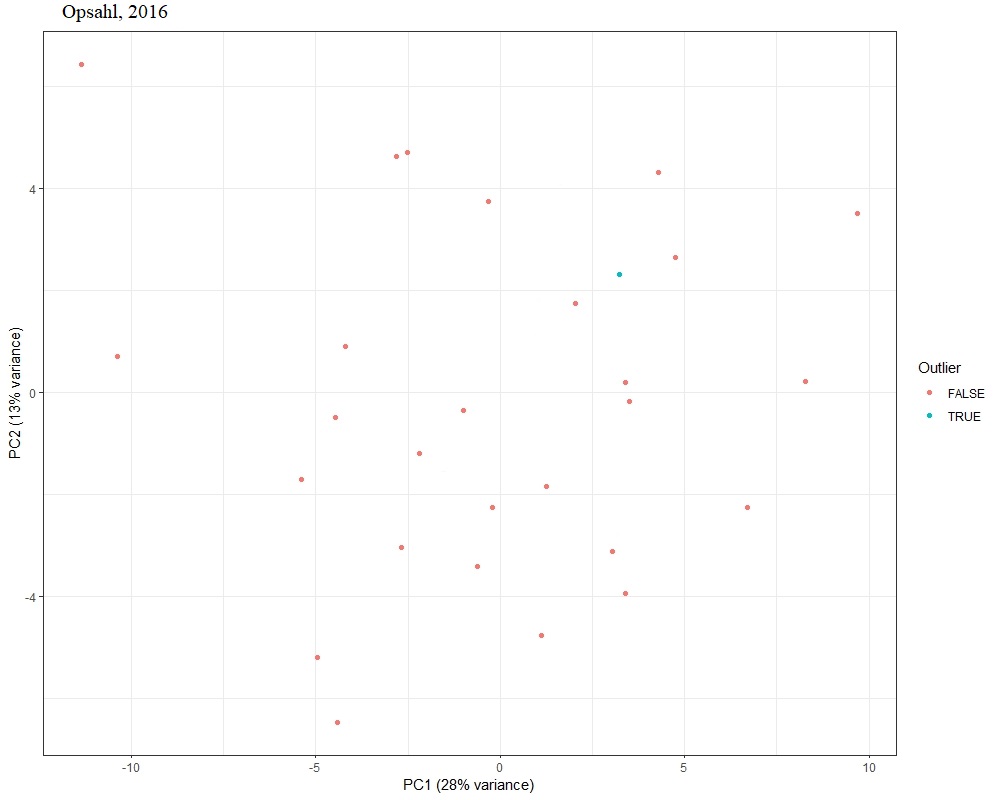

Supplement: Supplementary file 1 [file ijms-26-06171-s001.zip › Figure-S1-C.jpeg]

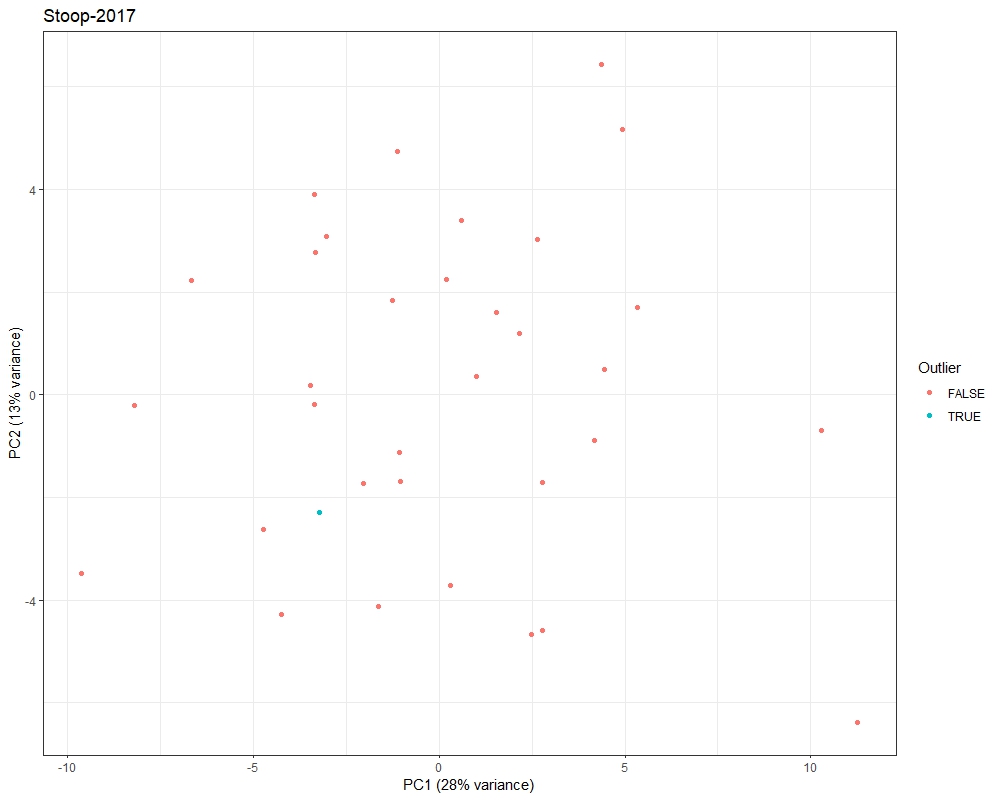

Supplement: Supplementary file 1 [file ijms-26-06171-s001.zip › Figure-S1-D.jpeg]

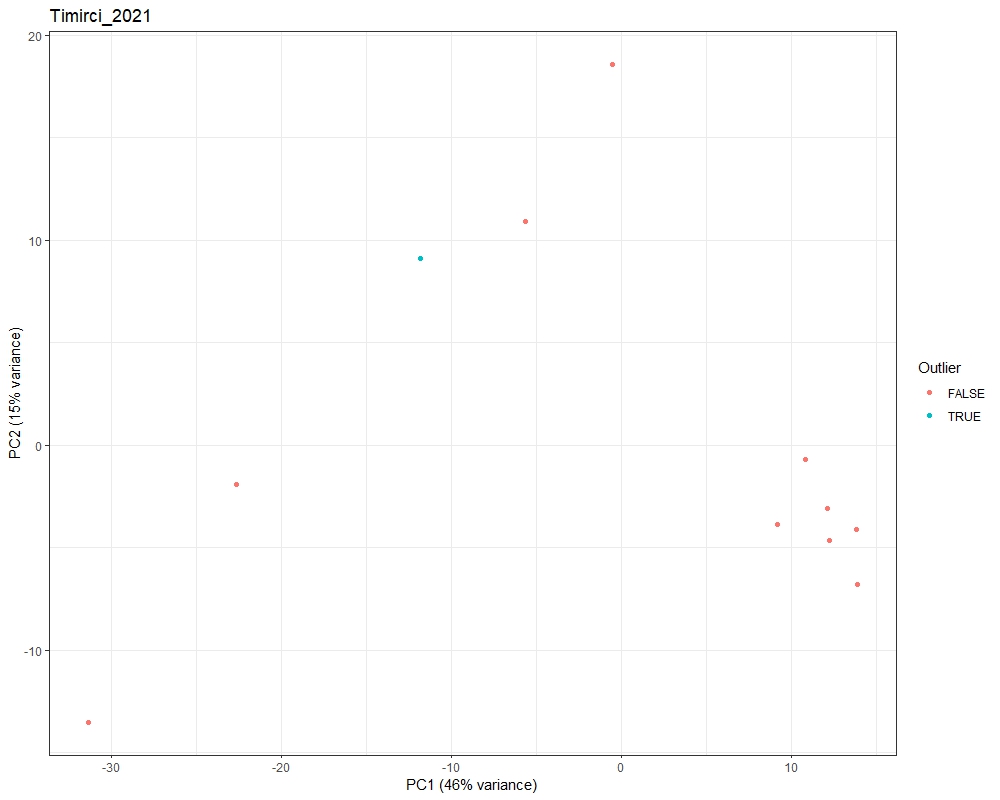

Supplement: Supplementary file 1 [file ijms-26-06171-s001.zip › Figure-S1-E.jpeg]

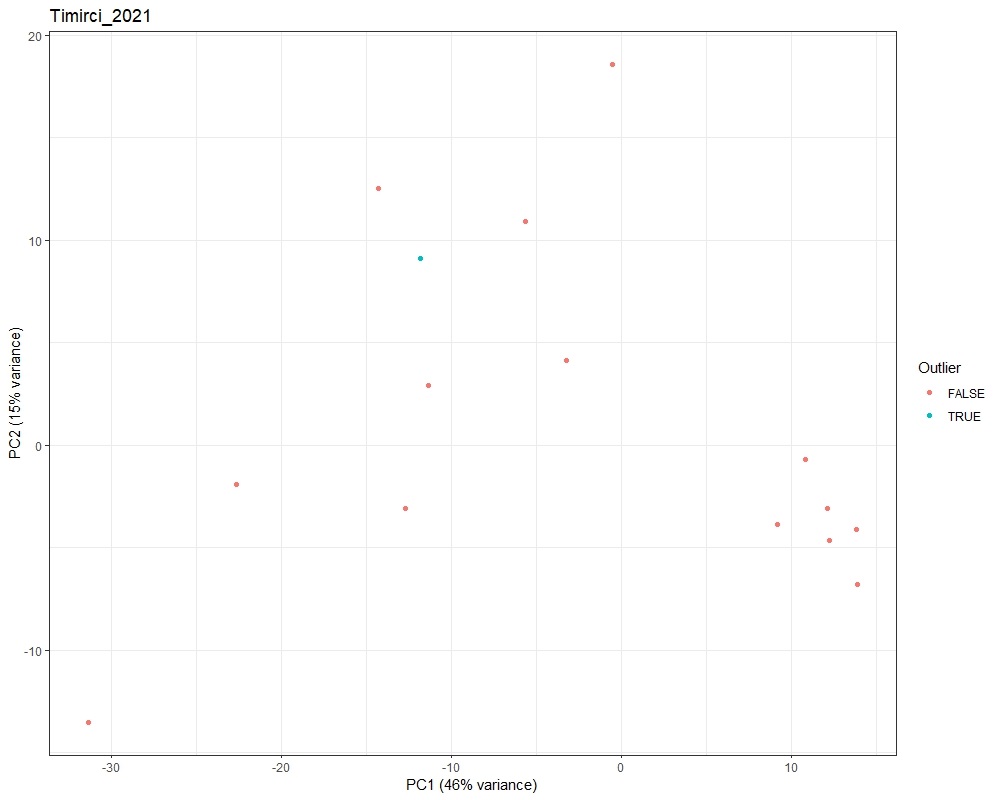

Supplement: Supplementary file 1 [file ijms-26-06171-s001.zip › Figure-S1-F.jpeg]

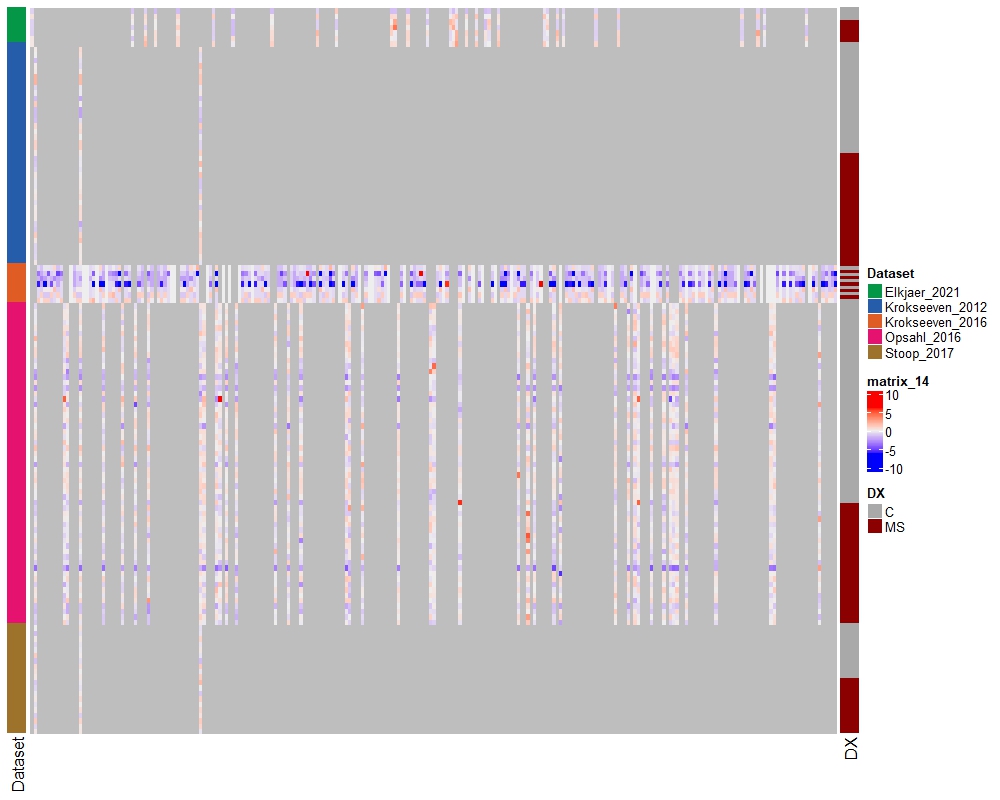

Supplement: Supplementary file 1 [file ijms-26-06171-s001.zip › Figure-S2-A.jpeg]

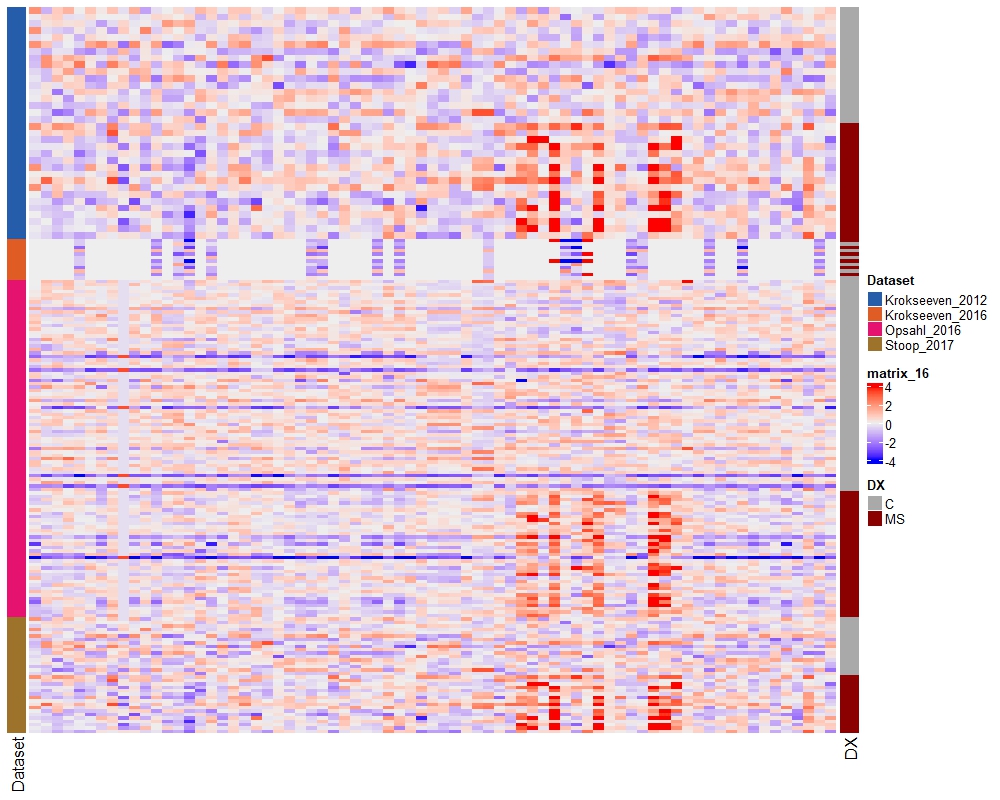

Supplement: Supplementary file 1 [file ijms-26-06171-s001.zip › Figure-S2-B.jpeg]
